# The Quality of Infrared Rotary Dried Terebinth (*Pistacia atlantica* L.)-Optimization and Prediction Approach Using Response Surface Methodology

**DOI:** 10.3390/molecules26071999

**Published:** 2021-04-01

**Authors:** Mohammad Kaveh, Yousef Abbaspour-Gilandeh, Ebrahim Taghinezhad, Dorota Witrowa-Rajchert, Małgorzata Nowacka

**Affiliations:** 1 Department of Biosystems Engineering, College of Agriculture and Natural Resources, University of Mohaghegh Ardabili, Ardabil 56199-11367, Iran; sirwankaweh@uma.ac.ir; 2 Department of Agricultural Technology Engineering, Moghan College of Agriculture and Natural Resources, University of Mohaghegh Ardabili, Ardabil 56199-11367, Iran; e.taghinezhad@uma.ac.ir; 3 Department of Food Engineering and Process Management, Institute of Food Sciences, Warsaw University of Life Sciences—SGGW, 02-776 Warsaw, Poland; dorota_witrowa_rajchert@sggw.edu.pl

**Keywords:** terebinth, color, shrinkage, rehydration rate, total phenolic compounds, antioxidant activity, infrared rotary drying

## Abstract

Most agricultural products are harvested with a moisture content that is not suitable for storage. Therefore, the products are subjected to a drying process to prevent spoilage. This study evaluates an infrared rotary dryer (IRRD) with three levels of infrared power (250, 500, and 750 W) and three levels of rotation speed (5, 10, and 15 rpm) to dry terebinth. Response surface methodology (RSM) was used to illustrate and optimize the interaction between the independent variables (infrared power and rotation speed) and the response variables (drying time, moisture diffusivity, shrinkage, color change, rehydration rate, total phenolic content, and antioxidant activity). As infrared power and rotation speed increased, drying time, rehydration rate, antioxidant activity, and total phenolic content decreased, while the other parameters were increased. According to the results, the optimum drying conditions of terebinth were determined in the IRRD at an infrared power of 250 W and drum rotation speed of 5 rpm. The optimum values of the response variables were 49.5 min for drying time, 8.27 × 10^−9^ m^2^/s for effective moisture diffusivity, 2.26 for lightness, 21.60 for total color changes, 34.75% for shrinkage, 2.4 for rehydration rate, 124.76 mg GAE/g d.m. for total phenolic content and 81% for antioxidant activity.

## 1. Introduction

Terebinth (*Pistacia atlantica*) is a common food in Western Iran (Kurdistan, Kermanshah, Ilam, and West Azerbaijan provinces). It is a small fruit with a sour taste, which is a source of anthocyanin, total phenolic, and flavonoid compounds [1,2]. Furthermore, terebinth is an excellent source of protein, vitamins (A, B, and D), minerals, nutrients, and carbohydrates [3]. It also contains flavonoids, fatty acids and triglycerides, oleoresin, and essential oils [4]. In medicine, terebinth is a very powerful natural source for eliminating cancer and plays an important role in the treatment of chronic inflammatory bowel disease [5]. However, due to its seasonal and perishable nature, drying terebinth is a useful method to extend its shelf life.

Drying is used to reduce the microbial and enzymatic activity and the rate of chemical reactions of agricultural products in order to increase the shelf life of the product [6,7]. Over the past decade, researchers have investigated numerous methods to reduce the time of the drying process [8] as well as maintain food quality [9]. In the hot air drying process, due to the low thermal conductivity of the product, the rate of heat transfer to the internal parts is low. Thus, the time required for the hot air drying process is much higher than for other dryers [10]. Additionally, prolonged exposure to high temperatures significantly degrades the quality of the product [11,12,13]. Therefore, quick drying techniques with an additional source of power, such as infrared, microwave, or a combination of the techniques with a rotary drum, can be used for agricultural products to reduce the process time and increase the quality of the product [8,11].

The quality of the dried product has an important impact on consumer acceptance [14,15]. The first parameter which is evaluated by consumers is the color of the dried plant material. It is desirable that the color of the material being dried is as similar as possible to the fresh tissue [16]. Additionally, fruits and vegetables are important in the human diet due to containing many vitamins, minerals, bioactive compounds, and antioxidants [17]; however, during drying, the valuable compounds as well as color may be degraded. Still, the unfavorable changes that occur during the drying process can be limited through the appropriate selection of processing parameters and relevant pretreatment of raw materials [12,16,18,19].

Infrared radiation is a form of an electromagnetic wave caused by thermal vibrations of molecules, and conversely, absorption of the waves causes thermal vibration of the molecules [20]. The high heat flux is an advantage of this type of dryer over hot air dryers. Such a high heat flux within the product creates a high temperature difference and can consequently impair the quality of the product. For this purpose, these waves are used for thin-layer products. This process is faster in comparison to hot air drying, which results in the better maintenance of bioactive compounds, while the process cost is reduced [13]. 

The optimization of drying conditions is necessary to reduce drying time, which is usually related to increased product quality. Among the methods for optimization of the drying parameters is response surface methodology (RSM). This is a set of statistical techniques of data collection that can optimize the effect of factors affecting response variables in a process using mathematical and statistical models [21]. Fealekari and Chayjan [22] used RSM to optimize specific the energy consumption, color, shrinkage, and moisture diffusivity of Persian shallot in an infrared–hot air dryer, and the optimum point for the parameters were temperature of drying at 70 °C, thickness of 2 mm, and infrared power from 772 to 1050 W, while for drying mushrooms in an infrared–hot air dryer, the best parameters using RSM were set for 839 W infrared power, temperature of 90 °C, and 90% of air recirculation at 1 m/s airflow [23]. However, the optimization of industrial processes requires the simultaneous optimization of several objective functions. Some processes may aim to maximize responses and others to minimize responses [6]. 

Considering the advantages of infrared dryers and the promising combination with the rotary drum dryer, this study aimed to model the response variables (qualitative, nutritional, and thermodynamic properties) of terebinth under the influence of independent variables (infrared power and rotation speed) in an infrared rotary dryer (IRRD) as well as determine the optimal conditions during terebinth drying using response surface methodology. Due to the colorants belonging to different groups of chemical substances, in dried terebinth, the total polyphenols content, as well as the antioxidant activity, was studied.

## 2. Results and Discussion

### 2.1. Optimization Parameters of IRRD Drying of Terebinth

In Table 1, the fitting effect of different levels of infrared power and rotary rotation speed on independent parameters was presented, which will be discussed in the following subchapters. The model’s fitting was evaluated on the basis of the coefficient of determination (R^2^), adjusted R^2^, predicted R^2^ and coefficient of variation (CV). All the R^2^ values were high (>0.98) for all the responses, which means that the response surface methodology models were suitable. Furthermore, the coefficient of variation for almost all parameters was below 5%, with one exception for lightness (12.6%). Thus, this means that the results demonstrated good accuracy and precision with the reliability of experiments. 

### 2.2. Drying Time and Effective Moisture Diffusivity of IRRD Dried Terebinth

**Figure 1:**
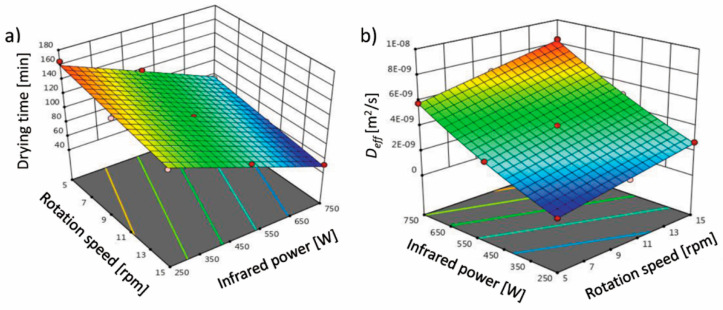
Effect of the infrared power and rotation speed on (**a**) drying time and (**b**) effective moisture diffusivity (*D_eff_*) of the terebinth dried under an infrared rotary dryer (IRRD).

**Figure 2:**
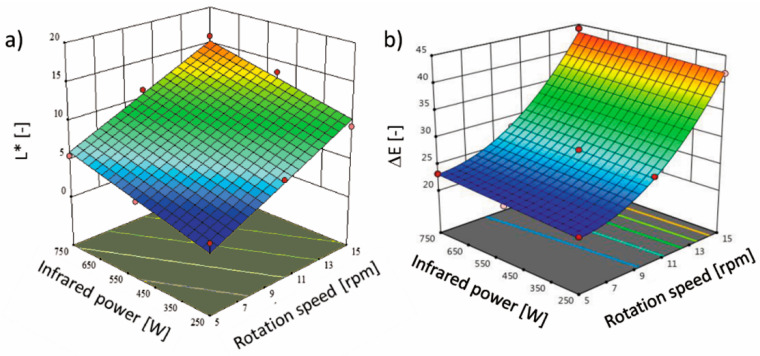
Effect of the infrared power and rotation speed on (**a**) lightness (L*) and (**b**) total color change in the terebinth dried under an IRRD.

Using infrared drying impact, the drying time is shorter even 47% in comparison to convective drying [24]. Figure 1 shows the curve of the impact level of infrared power and drum rotation speed on the drying time and effective moisture diffusivity (*D_eff_*) of terebinth. According to the results in Table 1, it was found that the linear effects of infrared power and rotation speed on the drying time of terebinth are significant (*p* < 0.05). The drying time of terebinth is strongly influenced by the infrared power (higher coefficient for B: infrared power than A: rotation speed in the equation in Table 1). As the infrared power and the rotation speed increased, the drying time of terebinth decreased (Figure 2a). The reduction in drying time with increasing infrared power may be due to the faster removal of moisture from the product at high infrared powers. This is because increasing the output infrared power increases the temperature in the chamber and the intensity of the infrared rays, which further reduces the moisture content of the terebinth. Thus, the higher the infrared power, the greater the mass and heat transfer, and the greater the reduction in the moisture content of terebinth. This is in agreement with other researchers’ results [23,25,26], which show that the drying time significantly decreases by increasing the infrared power. The drying time is also reduced by increasing the mesh rotation speed. The reduction in the processing time by increasing the rotation speed may be due to the rapid removal of moisture from the product. The reduction in the processing time by increasing the drum rotation speed has been reported for green peas [27], chilies [28], and pistachios [29].

Additionally, the independent variables, infrared power and rotation speed, have an effect on *D_eff_*. The high value of R^2^ represents the suitability of the fitted models. The effect on *D_eff_* was significant according to the quadratic equation (*p* < 0.05). The positive sign of the regression coefficients estimated (R^2^, Table 1) indicates the direct influence on the amount of *D_eff_*. Figure 1b shows the interaction between the infrared power and rotation speed and the *D_eff_* of the terebinth samples dried in the IRRD using response surface methodology. The maximum *D_eff_* (8.34 × 10^−9^ m^2^/s) was obtained at an infrared power of 750 W and rotation speed of 15 rpm, and the minimum *D_eff_* (7.78 × 10^−10^ m^2^/s) was obtained at an infrared power of 250 W and rotation speed of 5 rpm. The effect of infrared power on *D_eff_* was such that an increase in infrared power (from 250 to 750 W) increased *D_eff_* due to greater mass transfer. When the samples were dried at high infrared powers, the increase in thermal energy increased the activity of the water molecules and ultimately the *D_eff_* [30]. These results are consistent with the reports of other researchers on black carrots [31], wormwood leaves [32], and hazelnut [33]. In biological products, the penetration of infrared power into the product causes the water molecules to vibrate. In this case, the molecules require less energy to move in the porous products. Therefore, increasing the infrared power increased the *D_eff_*. Figure 1b presents that increasing the drum rotation speed also increased the *D_eff_*, which can be attributed to the reduced drying time. For drying green peas in a microwave-hot air rotary drum dryer, Kaveh and Abbaspur Gilandeh [27] showed that increasing the drum rotation speed increases *D_eff_*.

### 2.3. Physical Properties (Color, Shrinkage, and Rehydration Rate) of IRRD Dried Terebinth

The infrared drying of terebinth influenced the physical properties. Color is a crucial factor [34]. The L* parameter of fresh terebinth is 1.75 ± 0.38, and the drying caused an increase in the terebinth lightness. After drying, the L* values were in the range of 2.26 (5 rpm and 250 W) and 15.93 (15 rpm and 750 W). Figure 2a shows the interaction of rotary rotation speed and infrared power on the L* parameter of dried terebinth. With increasing the infrared power and rotary rotation speed, an increase in the L* parameter of dried terebinth took place. Table 1 shows that infrared power and drum rotation speed have a significant (*p* < 0.05) linear effect on lightness. The color changes are the results of the formation of a brown pigment through the Millard reaction during the drying process as well as the decomposition of the pigments [34]. Furthermore, the lighter color might be related to the method of the measurement, where the reflection is different for wet and porosity material [35]. Thus, usually, the porous surface results in a lighter color. In the conducted study, the higher lightness of the sample is probably connected with the shorter drying time when the higher infrared power and rotation speed were used. However, the temperature of the drying also has an impact on the plant tissue color, and with the increasing temperature of the material during infrared drying, the lightness decreases [34]. Furthermore, the color and taste of processed plant products are influenced by many compounds present in the material, e.g., polyphenolic compounds [36].

Additionally, the infrared power and drum rotation speed have a significant (*p* < 0.05) effect on the total color change (ΔE) of dried terebinth according to a quadratic equation (Table 1). ΔE was calculated in comparison to fresh fruits. The two variables, infrared power and rotation speed, had a profound effect on the color change, but infrared power was the most influential factor in terms of the ΔE of the dried terebinth. The values of R^2^, Pre-R^2^, Adj-R^2^, and CV for color change were found to be 0.9980%, 0.9967%, 0.9931%, and 1.69%, respectively. As shown in Figure 2b, the ΔE increased with increasing infrared power and rotation speed. The lowest and highest values of ΔE change were determined as 21.81 and 44.19, respectively. The ΔE values were high, which means that the changes in the L*, a* and b* parameters were significant. The value of total color change when it is higher than five demonstrates significant changes between the sample and untreated sample, which is recognized by the inexperienced observer [37]. The results showed that the ΔE of terebinth at low infrared powers and rotation speeds was closer to the values of fresh samples. Kayran and Doymaz [38] reported that the ΔE of infrared dried carrot slices increased at different infrared powers ranging from 62 to 125 W, which was attributed to pigment degradation or non-enzymatic browning during the drying process. Kaveh et al. [39] reported that the ΔE of terebinth increased with increasing infrared power (from 500 to 1500 W) in a microwave–infrared–hot air dryer, which was due to pigment degradation or non-enzymatic browning. 

**Figure 3:**
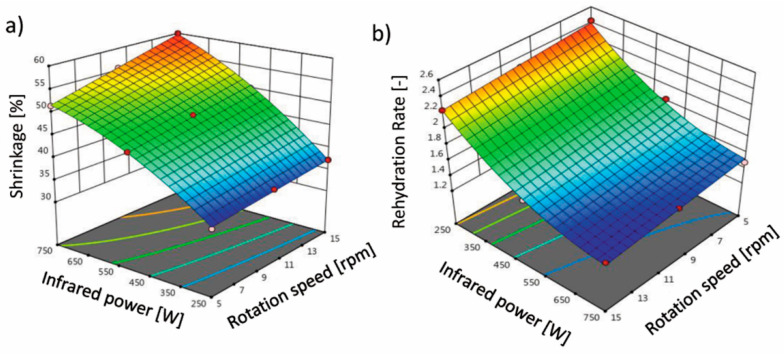
Effect of the infrared power and rotation speed on (**a**) shrinkage and (**b**) rehydration rate of the terebinth dried under an IRRD.

The shorter drying time causes lower shrinkage and better rehydration and hygroscopic properties [24]. According to Table 1, the effect of the variables, rotation speed and infrared power, on the percentage of shrinkage (S_b_) was significant (*p* < 0.05) and quadratic. This trend is evident in the response surface (Figure 3a), confirming the significance of the linear coefficients of the model. It can be noted that the highest S_b_ was 59.99% at a 750 W power and 5 rpm rotation speed, while the lowest S_b_ was recorded at a 250 W power and 5 rpm rotation speed. By increasing the rotation speed and infrared power, the percentage of S_b_ increases. The results of this study are in good agreement with the observations of Ghavidelan and Chayjan [33]. This may be due to the rapid release of water (moisture) from the terebinth tissue with increasing infrared power and rotation speed. In general, heating and moisture removal causes stresses in the cellular structure of the food, resulting in the deformation and reduction in the size of the product. Fruits and vegetables have a high moisture content, and their structure changes due to S_b_. During the drying process, water is removed from the cell, the tension that the water exerts on the cell wall is reduced, and this reduction in tension causes the product to shrink [40]. Furthermore, shrinkage is associated with an increase in the density of the sample, which, on the other hand, results in less porosity [11,24]. Ruhanian and Movagharnejad [41] investigated the thin layer drying of potato in a combined infrared–hot air dryer and found that shrinkage of the samples increased with increasing infrared power. They stated that increasing the infrared power increased the amount of heat given to the food. Therefore, greater moisture gradients occur in the samples, resulting in increased internal stress and consequently increased shrinkage. 

For dried material, the rehydration properties are important. These properties are related to the adsorption of water by the dried material. The higher the rehydration rate, the better, which means that the structure of the tissue has not been damaged, or small changes occur during the drying process [14]. The rehydration rate of the dried terebinth samples varied from 1.41 to 2.43. Table 1 shows the equation coefficients obtained from the models fitted to the experimental data. The results showed that infrared power, rotation speed, and the interaction between infrared power and rotation speed had a statistically significant effect (*p* < 0.05). The CV, Pre-R^2^, Adj-R^2^, and R^2^ values of 2.38, 0.9736, 0.9879, and 0.9925, respectively, were obtained by the model, and due to the high R^2^ values, the quadratic model was reported as the best model. It was found from the proposed model that the RR changes significantly with rotation speed and infrared power and increases when rotation speed and infrared power are reduced. Figure 3b shows the effects of infrared power and rotation speed on the response variable (rehydration rate). Decreasing the amount of independent variables increased the RR. With decreasing the infrared power from 750 to 250 W, the RR increased from 1.41 to 2.43. This may be because the conductivity and moisture gradient of the samples increase at higher powers, resulting in rapid drying of the product. This is mainly due to the higher mass transfer rate, which leads to a decrease in the RR. It is very likely to contribute to the acceleration of shrinkage due to the inability of the terebinth texture to maintain its network structure [42]. For drying savory leaves in an infrared–hot air dryer, Darvishi et al. [6] showed that the highest RR was obtained at the lowest infrared power, while the lowest RR was obtained at the highest infrared power, which is consistent with the results of this study. In Figure 2b, the RR was reduced with increasing rotation speed while keeping the infrared power constant. The maximum RR (2.43) was obtained at an infrared power of 250 W and rotation speed of 5 rpm, and the lowest RR (1.41) was obtained at an infrared power of 750 W and rotation speed of 15 rpm. 

### 2.4. Bioactive Compounds (Total Phenolic Compounds and Antioxidant Activity) of IRRD Dried Terebinth

**Figure 4:**
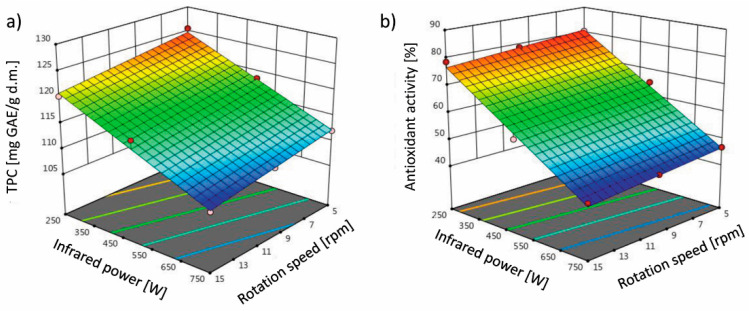
Effect of the infrared power and rotation speed on (**a**) total phenolic compounds (TPC) and (**b**) antioxidant activity (with DPPH) of the terebinth dried under an IRRD.

Evaporation of the water causes the material matrix to dry and the concentration of water-soluble substances to increase. The concentration of ingredients may accelerate the course of chemical and enzymatic reactions in the product [43]. According to Table 1, the linear model is suitable for predicting total phenolic compounds (TPC) and antioxidant activity (AA) under the influence of the variables under study (infrared power and rotation speed). The independent effect of infrared power and mesh rotation speed on TPC and AA was significant (*p* < 0.05). Figure 4 shows the curve of change in the response surface for the TPC (Figure 4a) and AA (Figure 4b) of terebinth after drying. The TPC and AA obtained in this study under different drying conditions ranged from 108.05 to 125.57 mg GAE/g d.m. and from 43.21% to 80.52%, respectively. It is very important to maintain product quality after the drying process. Some factors, such as the drying method (type of dryer, temperature, infrared power, and rotation speed) as well as pretreatments, affect the TPC and AA properties of the dried products [7,18,44]. The TPC and AA decreased with increasing the infrared power from 250 to 750 W and the rotation speed from 5 to 15 rpm. This is because phenolic compounds are very active and have unstable chemical properties. The high infrared power and rotation speed can accelerate the reaction rate of thermal degradation of phenolic compounds, thus reducing TPC and AA [45]. In the dried foods, there is a direct relationship between TPC and AA properties [46]. Therefore, the reduction in TPC also reduces AA.

Zeng et al. [34] investigated the effect of different infrared powers on the TPC of kiwifruit. They reported that the amount of TPC of dried kiwifruit at lower infrared powers was lower than at higher powers, which was attributed to the reduced drying time from 570 to 210 min with increasing infrared power. This is due to the shorter oxidation reaction time of TPC at higher infrared powers (lower drying times). As mentioned before, the AA of dried terebinth increased with the reduction in infrared power from 750 to 250 W. This may be due to the increased AA of polyphenolic compounds in the intermediate oxidation phases compared to the early oxidation phases or to the formation and aggregation of melanoidins as a result of the non-enzymatic Maillard browning reaction at lower temperatures, which increases AA [46]. The use of higher powers causes the degradation of anthocyanins and polyphenolic compounds [47]. 

### 2.5. Optimization of Terebinth Drying Process in the IRRD 

For the experimental data obtained from the IRRD, the utility function method presented the optimal conditions with a desirability of 0.98 (using Design Expert v. 10). The software determined the optimal conditions of the independent variables based on the goal of maximizing or minimizing the response variables. In this study, the optimal conditions were based on the maximum values of the response variables, rehydration ratio, total phenolic content, and antioxidant activity, and the minimum values of the response variables, drying time, shrinkage, total color change (∆E), and lightness. The solution proposed by the utility function showed that the optimal conditions for drying terebinth in the infrared rotary dryer were obtained at an infrared power of 250 W and rotation speed of 5 rpm. In the optimal points, the values of the response variables drying time, lightness, ΔE, shrinkage, rehydration rate, total phenolic content, and antioxidant activity were determined as 49.5 min, 2.26, 21.60, 34.75%, 2.4, 124.76 mg GAE/g d.m., and 81%, respectively, with the desirability of 0.98. 

Numerous studies have been conducted to determine the optimum conditions for agricultural products. For example, Afzali et al. [23] used response surface methodology to determine the optimal conditions of dried mushroom slices. Temperature, infrared power, and air recirculation were selected as the independent variables, and drying time, exergy loss, and exergy efficiency as the dependent variables. The optimal conditions for drying mushroom slices were reported using the thin layer method, air temperature of 90 °C, infrared power of 839 W, and 90% recirculation. However, Darvishi et al. [6] reported the optimal conditions for savory leaves in a combined infrared–hot air dryer at an air temperature of 40-60 °C, air flow rate of 0.5–1.5 m/s, and infrared power of 0.181–0.253 W/cm^2^ to minimize drying time, color change, and specific energy consumption and maximize rehydration rate. They showed that the optimal points for drying time, total color change, rehydration rate, and specific energy consumption were determined as 29.83 min, 14.05, 2.94, and 10.50 MJ/kg, respectively. 

## 3. Materials and Methods

### 3.1. Preparation of Terebinth

The terebinth (var. kurdica) was obtained from the forests of Sardasht in West Azerbaijan province, Iran. The samples were cleaned manually, and all broken, defective fruits, as well as external particles, were removed. The material was stored in polyethylene nylon bags in a laboratory refrigerator at 4 °C until processing (around a week). The moisture content of terebinth was 3.85 ± 0.5% (d.m.%) and determined by oven drying (Memmert, UFB 500, Germany) of about 10 g of samples at 70 ± 2 °C for 24 h until reaching a constant mass [48]. 

### 3.2. Infrared Rotary Drying of Terebinth

**Figure 5:**
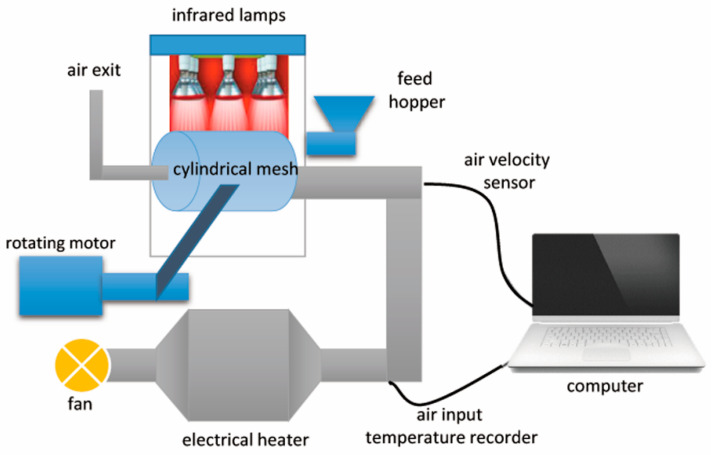
Scheme of infrared rotary dryer (IRRD).

A laboratory-scale infrared rotary dryer (IRRD) was used to perform the experiments (Figure 5). The dryer has a rectangular tank with dimensions 120 × 10 × 130 cm made of stainless steel with a thickness of 2 mm. A cylindrical mesh (drum) is placed inside the rectangular tank. To rotate the drum, two rollers are used at both ends of the chamber. The rollers rotate by a gearbox (VF 861/100). The gearbox speed is adjusted using an inverter (LS, Korea). Four infrared lamps each with a power of 250 W were used inside and above the dryer chamber to generate the infrared radiation. According to the experiment conditions (three infrared power levels), a maximum of three infrared lamps with a power of 750 W were used to generate the infrared power. 

The dryer was turned on 30 min before the start of each experiment to ensure stable and uniform conditions at the beginning of drying. The experiments were performed at three levels of infrared power (250, 500, and 750 W) and three levels of rotation speed (5, 10, and 15 rpm) in three replications. These parameters were chosen on preliminary studies. One hour before the start of the process, the required amount of terebinth samples was removed from the refrigerator to reach equilibrium with the ambient temperature. The drying process was finished after reaching 0.2 kg H_2_O/kg d.m. of the moisture content of the sample. The weight of the samples was measured using a balance (GF-600, Japan) with a precision of ±0.001 g. 

### 3.3. Drying Kinetics of IRRD Dried Terebinth 

The IRRD drying kinetics of terebinth were plotted as a function of dimensionless moisture ratio (MR) during the drying process. The MR was calculated according to the initial moisture content (M_o_), the equilibrium moisture content (M_e_), and the moisture content of the product at any time during drying (M_t_) using Equation (1) [49]:

MR = (M_t_ − M_e_)/(M_o_ − M_e_), (1)

Moreover, Fick’s second law was used to calculate *D_eff_*. Due to the sphericity of terebinth, it was assumed that the distribution of moisture in the product mass is uniform [50]. 

### 3.4. Physical and Chemical Properties of IRRD Dried Terebinth

#### 3.4.1. Shrinkage of IRRD Dried Terebinth 

The volume change method was used to determine the shrinkage of terebinth. For this purpose, 4 to 5 terebinths of each sample were placed in a graduated cylinder containing a certain amount of toluene, and the sample volume was determined based on the change made in volume. The measurement was conducted in three repetitions. The percentage of shrinkage (S_b_) was then calculated with the following equation [51]:

S_b_ = ((φ_i_ − φ_f_)/φ_i_) × 100, (2)

where φ_i_ is initial volume (cm^3^); φ_f_ is final volume (cm^3^).

#### 3.4.2. Color of IRRD Dried Terebinth

A colorimeter (portable colorimeter, HP-200, China) was used to measure the color of the samples before and after drying. The CIE L*a*b* system was used for measurement with the light source as D65, standard observer 2°, and measurement geometry de: 8°. Before measurement, the calibration of the device was performed with white and black tiles. For each experiment, the color measurement was made for 20 terebinth fruits, which were averaged and finally compared with the data of the fresh samples. The measurement was conducted in 10 repetitions. The lightness (L*) shows a spectrum from white to black with a range from 0 to 100, a* shows a spectrum from green to red with a range from −60 to +60, and b* shows a spectrum from blue to yellow with a range from −60 to +60. The total color changes (ΔE) of terebinth, in comparison to fresh fruits, were determined using the following equation [14].

ΔE = ((ΔL*)^2^ + (Δa*)^2^ + (Δb*)^2^)^0.5^, (3)

#### 3.4.3. Rehydration Rate (RR) of IRRD Dried Terebinth

To measure the rehydration rate, 4–5 g of the terebinth samples was added to 100 mL of distilled water. The samples were taken out of the water every half hour, dried with absorbent paper, and weighed until they reached a constant weight. The measurement was conducted in 3 repetitions. The rehydration rate of the samples was calculated by dividing the weight of the sample after rehydration (W_r_) per initial weight of the dried sample (W_b_) using the following equation [6].

RR = W_r_/W_b_, (4)

#### 3.4.4. Total Phenolic Content (TPC) and Antioxidant Activity (AA) of IRRD Dried Terebinth

To the 5 g of powdered dried samples was added 100 mL of 80% methanol solution and shaken at room temperature for 24 h; then, the supernatant of the centrifuged solutions was used to measure the factors. Total phenolic content (TPC) was measured using the Folin–Ciocalteu method [52]. For this purpose, 20 μL of the extract and 2.5 mL of the Folin–Ciocalteu reagent were added (to prepare the reagent, the Folin–Ciocalteu solution was diluted with distilled water in a ratio of 1 to 15) and allowed to stand for 3 min after mixing until the reaction tooks place. Then, 3 mL of 20% sodium carbonate was added and made up to 50 mL with distilled water after one minute. The sample was stored in the dark for 1 h, and the absorbance at a wavelength of 765 nm was read in comparison to the control. The total phenolic content of the sample was determined using the standard curve, and the results were calculated as mg of gallic acid per gram of dry terebinth using the equation fitted to the standard curve. The analysis was conducted in three repetitions.

The antioxidant activity (AA) of terebinth was measured using the free radical scavenging capacity of DPPH (2,2-diphenyl-1-picrylhydrazyl), which was expressed as a percentage of inhibition [20]:

AA [%] = ((A_blank_ − A_sample_)/A_blank_) × 100, (5)

In this method, 500 μL of the extract prepared for total phenol was centrifuged with 500 μL of distilled water at 1000 rpm for five minutes. Then, 150 μL of the solution was transferred to the test tubes, and finally, 2925 μL of methanol solution (2 mL of 0.1M methanol solution of DPPH) was added. After vortexing for a few seconds, the absorbance of the obtained solution (*A_sample_*) and control samples (*A_blank_*) was read using the spectrophotometer (80, UV/VIS Double Beam) at a wavelength of 517 nm. The analysis was conducted in three repetitions.

### 3.5. Experimental Design and Statistical Analysis

In this study, response surface methodology (RSM) was used to investigate the relationship between the independent variables of drying air temperature at three levels of infrared power (250, 500, and 750 W) and rotation speed (5, 10, and 15 rpm), and the response (dependent) variables, including the drying time [min], moisture diffusivity [m^2^/s], color [-], shrinkage [%], rehydration rate [-], antioxidant activity [%], and total phenolic content [mg GAE/g d.m.], by the following second-order polynomial equation [21]:

Y = B_0_ + B_1_X_1_ + B_2_X_2_ + B_12_X_1_X_2_ + B_11_X_1_^2^ + B_22_X_2_^2^, (6)

where Y was the response function; B_0_, B_1_, B_2_, B_11_, B_22_, B_12_, were coefficients; X_1_ was the rotation speed; X_2_ was the infrared power. Fitting of response surfaces and optimization of the drying process were performed using Design-Expert software v. 10 with RSM. The statistically significance of the independent variables on the response variables was examined at a 95% confidence level (*p* < 0.05), and only the variables with a significant effect on the response variable were used in the proposed regression equation. 

The optimization parameters were conducted using RSM with a statistically significance of the independent variables on the response variables examined at a 95% confidence level (*p* < 0.05). Only the variables with a significant effect on the response variable were used in the proposed regression equation. The experiments were performed at different rotation speeds and different infrared powers to evaluate their effect on responses such as drying time [min], lightness [-], total color change [-], shrinkage [%], rehydration rate [-], total phenolic content [mg GAE/g d.m.], and antioxidant activity [%]. The optimal point of the process was determined according to the boundary conditions and objective functions using Table 2. The chosen criteria for RSM optimization were a short time of drying (minimum time) and the maximum value for *D_eff_*, rehydration rate, and bioactive compounds (TPC and AA), while for the lightness (L*), total color change, and shrinkage, the minimum was set.

## 4. Conclusions

This study evaluated the effect of the independent variables (infrared power and rotation speed) on the response variables (drying time, lightness, total color change, shrinkage, rehydration rate, total phenolic content, and antioxidant activity) using the historical data design to determine the optimum conditions for drying terebinth in the infrared rotary dryer. Three levels of infrared power (250, 500, and 750 W) and three levels of rotation speed (5, 10, and 15 rpm) were used to conduct the experiments. The results showed that infrared power is the main parameter and is more effective than other parameters in terms of quality and drying time. The optimum conditions for drying terebinth were proposed at an infrared power of 250 W and rotation speed of 5 rpm. The optimum values of the response variables were obtained as 49.5 min for drying time, 8.27 × 10^−9^ m^2^/s for *D_eff_*, for lightness 2.26 and 21.60 for ΔE, 34.75% for shrinkage, 2.4 for rehydration rate, while 124.76 mg GAE/g d.m. for total phenolic content and 81% for antioxidant activity. The optimization leads to reduce waste and reduces the number of experiments required for drying—in this study, for drying terebinth with the use of the infrared rotary dryer.

## Figures and Tables

**Table 1:** Fitting effect of different levels of infrared power and rotary rotation speed on independent parameters.

Parameter	Equation	R^2^	Adj R^2^	Pred R^2^	CV [%]
DT	+215 − 2.76 × A − 0.16 × B	0.9905	0.9874	0.9770	4.1
*D_eff_*	−1.51 × 10^−9^ + 1.55 × 10^−10^ × A + 4.08 × 10^−12^ × B + 1.22 × 10^−13^ × A × B + 5.35 × 10^−15^ × B^2^	0.9993	0.9986	0.9950	2.2
L*	−6.09 + 0.93 × A + 0.01 × B	0.9630	0.9507	0.9140	12.6
ΔE	+25.04 − 1.79 × A + 0.003 × B + 0.19 × A^2^	0.9980	0.9967	0.9931	1.7
S_b_	+19.86 − 0.03 × A + 0.06 × B + 0.001 × A × B − 0.00003 × B^2^	0.9986	0.9971	0.9910	1.0
RR	+3.44 − 0.016 × A − 0.004 × B + 0.000002 × B^2^	0.9925	0.9879	0.9736	2.4
TPC	+132.99 − 0.39 × A − 0.025 × B	0.9808	0.9744	0.9596	0.8
AA	+100.51 − 0.448 × A − 0.069 × B	0.9886	0.9848	0.9767	3.0

A: rotation speed (rpm); B: infrared power (W); R^2^: determination coefficient; CV: coefficient of variation.

**Table 2:** Boundary conditions of independent and dependent variables.

Parameter	Symbol	Unit	Category	Target	Min	Max
Infrared power	P	W	Input	In the range	250	750
Rotation speed	V	rpm	Input	In the range	0.5	1
Drying time	DT	min	Output	minimum	52	165
Moisture diffusivity	*D_eff_*	m^2^/s	Output	maximum	7.78 × 10^−10^	8.34 × 10^−9^
Lightness	L*	-	Output	minimum	2.26	15.93
Total color change	ΔE	-	Output	minimum	21.81	44.19
Shrinkage	S_b_	%	Output	minimum	34.38	59.99
Rehydration rate	RR	-	Output	maximum	1.40	2.43
Total phenolic content	TPC	mg GAE/g d.m.	Output	maximum	108.05	125.57
Antioxidant activity	AA	%	Output	maximum	43.21	80.52

## Data Availability

The data presented in this study are available on request from the corresponding author.

## Sample Availability

Samples of the compounds are not available from the authors.
